# Submicroscopic Malaria in Migrants from Sub-Saharan Africa, Spain

**DOI:** 10.3201/eid2502.180717

**Published:** 2019-02

**Authors:** Joaquín Pousibet-Puerto, Mª Teresa Cabezas-Fernández, Ana B. Lozano-Serrano, José Vázquez-Villegas, Manuel J. Soriano-Pérez, Isabel Cabeza-Barrera, José A. Cuenca-Gómez, Joaquín Salas-Coronas

**Affiliations:** Hospital de Poniente, El Ejido, Spain

**Keywords:** submicroscopic malaria, malaria, screening, co-infection, filariasis, hemoglobinopathies, vector-borne infections, parasites, migrants, Spain, Africa, sub-Saharan Africa

## Abstract

In a screening program, we detected submicroscopic malaria in 8.9% of recent migrants to Spain from sub-Saharan Africa. Hemoglobinopathies and filarial infection occurred more frequently in newly arrived migrants with submicroscopic malaria than in those without. Our findings could justify systematic screening in immigrants and recent travelers from malaria-endemic areas.

Submicroscopic malaria (SMM) is defined as low-density *Plasmodium* infection detected only by molecular methods (*1*). SMM only occasionally causes acute disease but can infect mosquitoes and contribute to transmission (*2*).

In malaria-endemic countries, SMM prevalence varies widely. It is highest in areas of low transmission, where SMM represents a large proportion of the malaria reservoir (*2*). In regions to which malaria is not endemic, such as Europe, SMM prevalence is unknown but might account for up to one third of imported malaria cases (*3*). In areas such as Spain, *Anopheles atroparvus* mosquitoes can transmit strains of *P. vivax* (*4*), and SMM patients can be a reservoir for malaria reintroduction. We explored the frequency of imported SMM by PCR testing of a selected population of migrants to Spain from sub-Saharan Africa and describe the epidemiologic characteristics and main laboratory findings for SMM patients.

## The Study

We conducted a retrospective observational study based on data obtained after the application of an SMM screening protocol in immigrant patients of sub-Saharan Africa origin seen at the Tropical Medicine Unit of the Poniente Hospital (El Ejido, Almeria, Spain) during October 2004–December 2016. This hospital’s protocol comprised a series of complementary tests to screen for imported diseases, including chest and abdominal radiographs; blood count; liver and renal function tests, iron metabolism tests; serologic screening for syphilis, HIV, hepatitis B virus, hepatitis C virus, *Strongyloides*, and *Schistosoma*; and screening for fecal parasites, urine parasites, and blood microfilariae. Finally, the hospital tested for hemoglobinopathies using high-performance liquid chromatography.

The study population comprised patients who had lived in Europe for <1 year (newly arrived migrants [NAM]) or who had visited their home country (i.e., visiting friends and relatives [VFR]) within the previous year who sought care for any reason other than patent malaria and were screened for SMM using the conventional nested multiplex malaria PCR. The nested multiplex malaria PCR can identify 4 human malaria species (*P. vivax*, *P. falciparum*, *P. ovale*, and *P. malariae*) in 2 consecutive multiplexing amplifications. The first reaction amplifies *Plasmodium* DNA from blood samples. The second reaction enables identification of the infecting species of *Plasmodium* (*5*). SMM was diagnosed when a patient had a positive malaria PCR result and a negative direct microscopic examination result, either a thin or a thick smear, and a negative rapid diagnostic test (SD. BIOLINE Malaria Ag P.f/Pan test, Abbott, https://www.alere.com). We excluded from the study patients <14 years of age and patients for whom no smear and/or rapid diagnostic test for malaria was available. All SMM patients were treated according to World Health Organization guidelines (*6*). 

We conducted 3 statistical analyses. First, we compared SMM patients with non-SMM patients. Then, within the SMM patient group, we compared those with and without filarial co-infection. We conducted a descriptive statistical analysis in which continuous variables were expressed as medians and interquartile ranges. Categorical variables were described as frequencies and percentages. We analyzed differences in continuous data between groups using nonparametric Mann-Whitney U test and used the Fisher exact test or χ^2^ test, as appropriate, to compare categorical data. Finally, we conducted an explanatory multivariate logistic regression analysis to evaluate possible risk factors predicting SMM in the study population. The model used variables with p<0.2 in the bivariate analysis and those that were clinically relevant. Variables were excluded from the logistic regression model based on likelihood ratio test results (*7*). Hosmer-Lemeshow test and the area under the receiver operating characteristic curve were used to validate the model. We conducted statistical analyses using STATA software version 12 (https://www.stata.com).

Of 2,719 sub-Saharan Africa patients seen, 370 (13.6%) were included in the study (Table 1). SMM was diagnosed in 33 (8.9%) patients, of whom 11 were VFRs and 22 were NAMs. The proportion of SMM was similar in both groups: 8.7% (11/126) for VFRs and 9.0% (22/244) for NAMs (p = 0.93). For SMM patients, time spent in Spain after leaving malaria-endemic areas was shorter for VFRs (2 months) than for NAMs (6 months) (p = 0.001).

**Table 1:** Epidemiologic characteristics and findings of laboratory blood tests of patients in a study of SMM in migrants from sub-Saharan Africa to Spain, October 2004–December 2016

Characteristic	All, N = 370	Non-SMM, n = 337	SMM, n = 33	p value
Age, y, median (IQR)	28 (13)	28 (12)	27 (14)	0.78
Sex, no. (%)				
M	309 (83.5)	283 (84.0)	26 (78.8)	0.44
F	61 (16.5)	54 (16.0)	7 (21.2)	
Country of origin, no. (%)				
Senegal	102 (27.6)	98 (29.1)	4 (12.1)	
Guinea Bissau	69 (18.6)	64 (19.0)	5 (15.2)	
Mali	61(16.5)	53 (15.7)	8 (24.2)	
Mauritania	28 (7.6)	28 (8.3)	0	
Equatorial Guinea	23 (6.2)	16 (4.8)	7 (21.2)	
Gambia	20 (5.4)	19 (5.6)	1 (3.0)	
Burkina-Faso	18 (4.9)	14 (4.2)	4 (12.1)	
Ghana	18 (4.9)	16 (4.8)	2 (6.1)	
Guinea-Conakry	16 (4.3)	16 (4.8)	0	
Nigeria	7 (1.9)	6 (1.8)	1 (3.0)	
Ivory Coast	5 (1.4)	4 (1.2)	1 (3.0)	
Cameroon	1 (0.3)	1 (0.3)	0	
Gabon	1 (0.3)	1 (0.3)	0	
Democratic Republic of the Congo	1 (0.3)	1 (0.3)	0	
Type of traveler, no. (%)				
NAM, <1 y of stay	244 (65.9)	222 (65.9)	22 (66.7)	
VFR, returned <1 y	126 (34.1)	115 (34.1)	11 (33.3)	0.93
Referring hospital department, no. (%)				
Primary care	279 (75.4)	255 (75.7)	24 (72.7)	
Emergency	27 (7.3)	23 (6.8)	4 (12.1)	
Internal medicine	17 (4.6)	16 (4.7)	1 (3.0)	
Other	47 (12.7)	43 (12.8)	4 (12.2)	
Main reason for referral, no. (%)				
Abdominal pain	107 (28.9)	102 (30.3)	5 (15.2)	
Viral hepatitis or liver test abnormalities	80 (21.6)	72 (21.4)	8 (24.2)	
Eosinophilia	53 (14.3)	43 (12.8)	10 (30.3)	
Pruritus or skin disorders	21 (5.7)	19 (5.6)	2 (6.1)	
Hematuria	9 (2.4)	9 (2.7)	0	
Median time to SMM screening since travel, mo. (IQR)				
Total	5 (5)	5 (5)	3 (4)	0,03
Newly arrived	6 (5)	5.5 (5)	6 (5)	0,66
VFR	4 (7)	4 (8)	2 (3)	<0.01
Malaria prophylaxis in VFRs, no. (%), n = 126				
No		59 (51.3)	5 (45.4)	0.79
Inadequate		7 (6.1)	1 (9.1)	
Yes		32 (27.8)	4 (36.4)	
Unknown†		17 (14.8)	1 (9.1)	
Baseline laboratory data, median (IQR)‡				
Hb, g/dL	14.85 (2.1)	14.9 (2.1)	14.6 (2)	0.22
Platelets, × 10^3^/μL	233.5 (83)	233 (83)	247 (71)	0.72
Total eosinophils/μL	280 (481)	270 (440)	440 (614)	0.12
Total bilirubin, mg/dL	0.66 (0.47)	0.62 (0.53)	0.72 (0.36)	0.71
Alanine aminotransferase, U/L	20 (17)	20 (17)	27 (7)	0.79
Aspartate aminotransferase, U/L	27 (11)	27 (12)	20 (12.5)	0.80
IgE, U/mL	299.46 (1033)	284.86 (986.31)	484 (923.83)	0.14
Structural hemoglobinopathies study, no. (%), n = 367 patients	85	71/334 (21.3)	14/33 (42.4)	0.01
Heterozygous Hb S		40	7	
Heterozygous Hb C		12	2	
Homozygous Hb C		1	3	
Thalassemia trait or α-thalassemia		13	1	
β-thalassemia minor		3	1	
Others		2		

*Hb, hemoglobin; IQR, interquartile range. NAM, newly arrived migrant; SMM, submicroscopic malaria; VFR, visiting friends and relatives. †Patients with unknown malaria prophylaxis were not included in the statistical analysis.  ‡Reference values: Hb 13.5–16.5 g/dL; platelets 130–450 × 1,000/μL; eosinophils 20–450/μL; total bilirubin 0.3–1.2 mg/dL; alanine aminotransferase 10–50 IU/L; aspartate aminotransferase 1–50 IU/L; IgE 0–100 IU/mL.

The *Plasmodium* species most frequently found was *P. falciparum* (26 [78.8%] patients), followed by *P. malariae* (4 [12.1%]), *P. ovale* (2 [6.1%]), and 1 mixed parasitization by *P. falciparum* and *P. malariae* (1 [3.0%]). Patients with and without SMM did not differ in baseline laboratory data, except for the presence of hemoglobinopathies, which occurred more frequently among SMM patients (42.4% vs. 21.3%; p = 0.01).

When we analyzed other associated infections (Table 2), we found an important difference between SMM and non-SMM patients regarding filarial co-infection. Filariasis was present in up to 24.2% of SMM patients but in only 5.3% of non-SMM patients (p<0.01). *Mansonella perstans* nematodes were responsible of all filarial infections; in addition, 3 patients were infected by *Loa loa* eyeworms.

**Table 2:** Co-infections in patients in a study of SMM in migrants from sub-Saharan Africa to Spain, October 2004–December 2016*

Co-infection	All, no. (%), N = 370	Non-SMM, no. (%), n = 337	SMM, no. (%), n = 33	p value
*Blastocystis hominis*	91 (24.6)	85 (25.2)	6 (18.2)	0.33
*Entamoeba hystolitica/dispar*	56 (15.1)	53 (15.7)	3 (9.1)	0.28
*Giardia lamblia*	25 (6.8)	24 (7.1)	1 (3.0)	0.35
*Strongyloides stercoralis*	73 (19.7)	67 (19.9)	6 (18.2)	0.75
Hookworms	39 (10.5)	37 (11.0)	2 (6.1)	0.36
*Trichuris trichiura*	11 (3.0)	10 (3.0)	1 (3.0)	0.99
*Ascaris lumbricoides*	10 (2.7)	8 (2.4)	2 (6.1)	0.23
Schistosomiasis	34 (9.2)	31 (9.2)	3 (9.1)	0.94
* S. haematobium*	21 (5.7)	19 (5.6)	2 (6.1)	0.95
* S. mansoni*	7 (1.9)	7 (2.1)	0	0.40
* S. intercalatum*	1 (0.3)	1 (0.3)	0	1
*Schistosoma* spp.	5 (1.4)	3 (0.9)	1 (3.0)	0.45
*Hymenolepis nana*	7 (1.9)	6 (1.8)	1 (3.0)	0.64
*Taenia* spp.	2 (0.5)	2 (0.6)	0	1
Filariae†	26 (7.0)	18 (5.3)	8 (24.2)	<0.01
* Mansonella perstans*	26 (7.0)	18 (5.3)	8 (24.2)	<0.01
* Loa loa*	3 (0.8)	1 (0.3)	2 (6.1)	0.02
Syphilis	39 (10.5)	33 (9.8)	6 (18.2)	0.12
Hepatitis B virus	111 (30)	98 (29.1)	13 (39.4)	0.17
Hepatitis C virus	5 (1.4)	4 (1.2)	1 (3.0)	0.37
HIV	2 (0.5)	1 (0.3)	1 (3.0)	0.17

*SMM, submicroscopic malaria.  †All 3 patients infected with *Loa loa* eyeworms were co-infected with *Mansonella perstans* nematodes.

**Figure 1:**
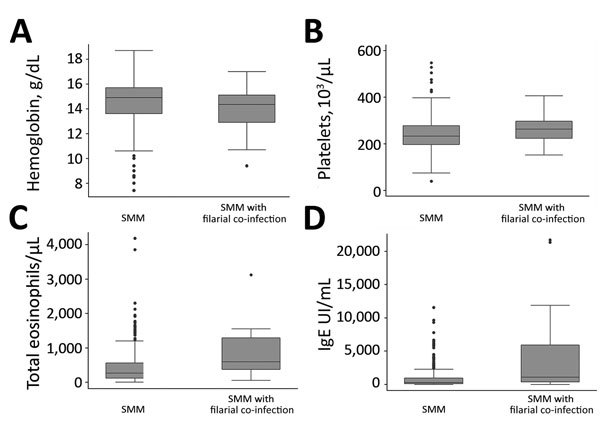
Differences in analytical values of blood tests among SMM patients with and without filarial co-infections, Spain, October 2004–December 2016. A) Hemoglobin; B) platelets; C) total eosinophils; D) IgE. Box and whiskers plot features are defined as follows: horizontal line within box is median, bottom line of box is 25th percentile, top line of box is 75th percentile, bottom whisker is quartile 1 – 1.5 interquartile range, top whisker is quartile 3 + 1.5 interquartile range, and dots are outliers. SMM, submicroscopic malaria.

Among SMM patients, all filariasis was found in NAMs. These co-infected patients had higher IgE values ​​(1,080 IU/mL vs. 293.7 IU/mL [reference 0–100 IU/mL]; p<0.01) and higher total eosinophil counts (601.5 cells/μL vs. 270 cells/μL [reference 20–450 cells/μL]; p = 0.01) than those who had only SMM. The co-infected group also tended to have higher platelet levels (Figure 1).

**Figure 2:**
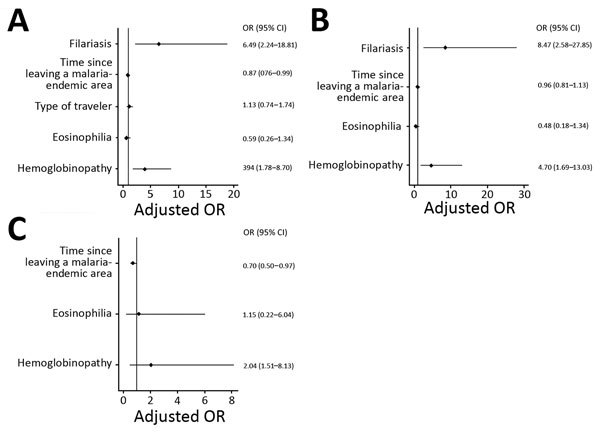
Multivariate logistic regression analysis for study of submicroscopic malaria in migrants from sub-Saharan Africa, Spain, October 2004–December 2016. A) All patients; B) newly arrived migrants; C) migrants visiting friends and family. OR, odds ratio.

Multivariate regression analysis applied to all 370 patients showed that having filarial infection increased the odds of having SMM by 6.49 and the existence of >1 hemoglobinopathies increased the odds by 3.93. Time after leaving a malaria-endemic area correlated inversely with risk for SMM (p = 0.038) (Figure 2). For NAMs, filariasis increased the risk for SMM by 8.47 and hemoglobinopathies by 4.70. For VFRs, however, the only risk factor was time since last visit to their home country (the shorter the time, the higher the risk).

## Conclusions

Screening for SMM in patients from sub-Saharan Africa in a reference unit in Spain showed a prevalence of 8.9%. The presence of filarial infection or hemoglobinopathies and a shorter time since leaving malaria-endemic areas were associated with a higher risk for SMM.

SMM is usually asymptomatic. Infrequently, it produces acute disease, especially in children (*8*). During pregnancy, SMM has been linked to maternal anemia and to low birth weight (*9*). SMM screening in risk groups, such as pregnant women and immunosuppressed persons (*10*), could therefore be of special interest.

Our study highlights 2 important differences between patients with and without SMM. First, the proportion of patients infected by filariasis was higher among SMM patients. In areas to which malaria is not endemic, Ramírez-Olivencia et al. also reported a greater number of filariasis among SMM patients than among patients with patent microscopic malaria (*3*). Nematodes can alter immune system response to concomitant infections, such as *Plasmodium* spp. Modulation of immune response produced by helminthoses, such as filariasis, might exert some protective effect against malaria, leading to lower parasitic loads, which in turn might translate into clinical protection against severe malaria (*11*,*12*).

The second disparity was the presence of hemoglobinopathies, a finding much more frequent among SMM patients that resulted in an SMM risk only for NAMs. Hemoglobinopathies exert a protective effect against severe malaria, favoring milder clinical manifestations and the existence of SMM (*11*,*14*).

The relatively high prevalence of imported SMM we found could justify implementation of systematic screening in immigrants and travelers who recently stayed in malaria-endemic areas, mainly for persons with risky conditions such as immunosuppression (especially those with HIV infection) and for pregnant women. The diagnosis and treatment of SMM also can prevent future reactivations and the existence of an occult malaria reservoir in countries to which it is not endemic. Our results suggest that the presence of filariasis, hemoglobinopathies, or both should also prompt a search for SMM because these patients are at higher risk.
